# The Australian Research Council Longevity Intervention (ARCLI) study protocol (ANZCTR12611000487910) addendum: neuroimaging and gut microbiota protocol

**DOI:** 10.1186/s12937-018-0428-9

**Published:** 2019-01-05

**Authors:** Tamara Simpson, Saurenne Deleuil, Nicole Echeverria, Mrudhula Komanduri, Helen Macpherson, Chao Suo, Shakuntla Gondalia, Masoumeh Tangestani Fard, Andrew Pipingas, Andrew Scholey, Con Stough

**Affiliations:** 1 Centre for Human Psychopharmacology, Swinburne University of Technology, PO Box 218, Hawthorn, Victoria, Melbourne, Australia; 2 Brain and Mental Health Laboratory, Monash Institute of Cognitive and Clinical Neurosciences, School of Psychological Sciences, Monash University, Melbourne, Australia; 3 Institute for Physical Activity and Nutrition Research, Deakin University, Melbourne, Australia

**Keywords:** Antioxidant, Neuroimaging, Pycnogenol, Bacopa, Glutathione, Cognition, Ageing, Magnetic resonance spectroscopy, Magnetic resonance imaging

## Abstract

**Background:**

The Australian Research Council Longevity Intervention (ARCLI) was designed to investigate the effects of two active supplements, Pycnogenol and *Bacopa monnieri* (CDRI08) on cognitive performance in a cohort of elderly participants. An additional antioxidant supplement has been included into the trial. A neuroimaging component has also been added to the ARCLI study to investigate the neurochemical biomarkers of oxidative stress in vivo, as well as structural and functional changes associated with ageing and oxidative stress*.* Faecal biomarkers of gut microflora will also be analysed to investigate if gut microbiota are associated with domains of cognition (e.g., attention, processing speed, memory), mood or other ARCLI outcome variables. The aim of this paper is to update the published methods of the ARCLI clinical trial before it is completed, and data analysis commences.

**Methods:**

ARCLI is a randomised, placebo controlled, double-blind, now 4-arm clinical trial including neuroimaging and gut microflora sub-studies. Along with the demographic, haematological, mood, cardiovascular and cognitive assessments described in the initial protocol, 80 eligible participants from the overall study pool of ~ 400 will be recruited into the neuroimaging study and undergo scans at baseline, 3 months and 12 months. Proton magnetic resonance spectroscopy, resting state functional connectivity and arterial spin labelled perfusion sequences are neuroimaging techniques included for each MRI visit in the study. Similarly, approximately 300 participants from the main study pool will be recruited to provide faecal samples at baseline, 3 months and 12 months so that the gut microbiome can be studied.

**Discussion:**

ARCLI is 12-month intervention study, currently underway with a group of older adults, investigating a range of outcomes and their association with ageing. The additional measurements in the ARCLI trial will further the understanding of the underlying mechanisms associated with healthy ageing and may provide insights into novel preventative therapeutic strategies for maintaining cognitive and brain health into old age.

**Trial registration:**

Australia and New Zealand Clinical Trials Register (ANZCTR): ACTRN12611000487970.

## Background and rationale

Studies have identified a link between alterations in brain neurochemicals and differences in cognitive function using the brain imaging technique of proton magnetic resonance spectroscopy (^1^H MRS) e.g. [1, 2]. Magnetic resonance spectroscopy (MRS) is a useful non-invasive technique to study in vivo neurometabolite concentrations. Understanding how nutritional supplements can modulate changes within the ageing brain will help to elucidate the biological processes involved in ageing. Using the neuroimaging modality of MRS may be a unique and sensitive measure of short and long-term in vivo intervention effects. The addition of an antioxidant rich supplement to the Australian Research Longevity Intervention (ARCLI) study will enable our group to extend our investigation to the cognitive, cardiovascular and general health benefits of antioxidants in an elderly cohort. The cognitive enhancing effects of vitamins and minerals have focussed on vitamin C, beta carotene and/or vitamin E [3] or a combination of nutrients in the form of a multivitamin [4, 5] with few studies using a complex antioxidant formula. A combined antioxidant formula may reduce the effects of oxidative stress, therefore improve cognitive function and assist older adults to live cognitively active lives. Additionally, *Bacopa monnieri* and Pycnogenol have been extensively studied in vitro to elucidate its antibacterial properties against various bacteria related to human health [6–8]. There is the possibility that these supplements have the capacity to modulate the gut microbiota and can affect the overall gut health and improve an individual’s general health status.

## Addition of an antioxidant combination supplement

The body employs complex interactions to negate oxidative stress. For instance, it utilises antioxidants which are either derived naturally within (endogenously) the body or provided externally (exogenously) through foods [9]. Antioxidants supplied through nutrition are the most important source to help endogenous antioxidants counteract oxidative stress reactions [10]. Nutritional antioxidants protect against increased oxidative stress by mainly scavenging free radicals to counteract free radicals, reduce peroxides and repair oxidized membranes, quench iron to reduce the creation of reactive oxygen species (ROS), and counteract ROS with short-chain fatty acids, via lipid metabolism [11].

Good nutrition from a varied diet and healthy vitamin and mineral status is integral to the biochemical processes that occur in the central nervous system, to maintain the brain’s structure and normal cognitive function [12]. In situations where the availability of endogenous antioxidants is insufficient to maintain cellular functions, exogenous antioxidants in the form of dietary supplements may be required to maintain optimal overall health and well-being [13]. The combined administration of alpha lipoic acid (LA) with other antioxidants such as acetyl-L-carnitine and Coenzyme Q10 (CoQ10), rather than the administration of LA in isolation, appears superior in attenuating cognitive and mitochondrial dysfunction [14]. The co-administration of a complex antioxidant blend containing 34 antioxidants when compared to placebo lowered serum homocysteine and improve declarative and non-declarative memory after four months in a group of older adults [15]. This suggests that there are synergistic benefits associated with the co-administration of different antioxidants. In a review of antioxidant use in human and animal studies, Kamat and colleagues [16] concluded that while animal models of antioxidant therapies were successful, the outcomes did not translate into human intervention research or clinical trials. One reason contributing to this lack of clarity in the literature is that ageing is likely to affect a myriad of metabolic sites whereby a single antioxidant supplement (eg. Vitamin C, Vitamin E, CoQ10) is unlikely to contribute to biochemical processes that form complex interactions. Targeting multiple metabolic sites may be a better method for effecting change in cognitive and health factors for older adults. There is a paucity of literature in ageing studies reporting findings of a blended antioxidant supplement. We therefore believe the efficacy of a combined antioxidant supplement to improve cognitive functioning and reduce the prevalence of risk factors associated with aging warrants further investigation, which is why a combined antioxidant supplement is now included in the ARCLI study.

## Rationale for the inclusion of neuroimaging

Neuroimaging will be conducted in the ARLCI trial with a subset of ~ 80 participants to explore in vivo neurochemical modes of action of *Bacopa monnieri* (CDR108), and Pycnogenol. As previously described in the main protocol, the results identified in human trials examining the effects of *Bacopa monnieri* and Pycnogenol on cardiovascular function and peripheral biochemical markers of inflammation and oxidation provide promising outcomes [17–20]. Studies like this inform possible peripheral biological mechanisms underpinning the actions of these supplements. However, to date, no studies have examined the in vivo brain effects using neuroimaging.

^1^HMRS is a magnetic resonance imaging (MRI) technique that will be used to measure the potential in vivo brain effects of these supplements by estimating regional concentrations of various neurometabolites. The metabolites most commonly measured with ^1^HMRS are N-aceytl aspartate (NAA), creatine (Cr), choline (Cho), myo-inositol (mI), and glutamate and glutamine (Glx) which represent underlying processes within the brain [21]. For example, NAA is considered to be a neural marker, Cr a marker of energy metabolism, Cho a marker of cellular turnover, mI a marker of inflammation and Glx a marker of metabolic activity [22]. Few studies have investigated the in vivo effects of natural supplement interventions on brain chemistry as measured with ^1^HMRS, thus these commonly measured metabolites will be investigated in the ARCLI study.

Recently, omega-3 fatty acid supplementation was reported to increase the chemical concentration of glutathione (GSH; an endogenous antioxidant) in the temporal lobe of first-episode psychosis patients, with this increase in GSH (the most abundant antioxidant in the body), correlating with improvement in negative symptoms [23]. GSH plays an integral role in protecting cells against reactive oxygen species [24] and is becoming a popular brain metabolite for researchers to investigate the integrity of antioxidant mechanisms and oxidative stress in vivo e.g. [25–27]. This popularity is most likely due to the negative effects of oxidative stress being implicated in chronic diseases and clinical disorders like depression [28], schizophrenia [29] and bipolar disorder [30] and neurodegenerative disorders like Alzheimer’s disease [31] where GSH as a biomarker is informative regarding the pathological neuronal changes occurring in the brain. GSH concentration will therefore be investigated in the ARCLI trial.

## Rationale for the examination of faecal biomarkers of gut microflora

Recently the human gut microbiota has become the subject of extensive research. The knowledge of the complex microbial community within the human intestinal tract and their functional capacity is rapidly growing. Gut microbiota influences human well-being, metabolism, physiology, nutrition, and immune function [32, 33]. Collectively studies now indicate that a disruption in the composition of the gastrointestinal microorganisms (or dysbiosis) can contribute to disease conditions such as diabetes [34], inflammatory bowel diseases [35], obesity [36], and malnutrition [37]; communicating through the central nervous system’s immune, neural and endocrine pathways [38–40]. As people age, the functionality of the immune system declines and can cause low level inflammation in the gut and in turn increase the permeability of the intestines [41]. Emerging literature suggests that the gut microbiota plays a role in the development of neurodegenerative disorders affecting cognition, particularly Alzheimer’s Disease [41, 42]. Therefore, it is apparent that any change to the microbial community can have a profound effect of both beneficial and harmful consequences for human health and cognition.

Interestingly in animal models, the role of gut microbiota has been studied using germ-free and specific pathogen free mice to investigate anxiety-like behaviour and to see how normal gut microbiota can modulate brain development and behaviour. Heijtz and colleagues [39] observed that normal gut microbiota affected normal brain development and behavioural functions in mice. They concluded that the alteration of proteins within the gut could lead to long term modification of synaptic transmission affecting motor control and anxiety-like behaviour in adult life. In their seminal study, Sudo and colleagues [43] identified a link between microbiota and hypothalamic-pituitary-adrenal reaction to restraint stress. Exaggerated corticosterone and adrenocorticotrophin response, indicators of increased stress reactivity, was found in these mice. This study was the first to identify that microbes can affect the neural network responsible for controlling stress responses and it became the motivation for neuroscientists to consider the importance of gut microbiota in central nervous system function. Gut microbiota analysis in the ARCLI study will be useful to investigate links between gut microbiota with mood and cognitive measures as well as seeing whether there are any alterations in gut microbial composition in response to nutraceutical supplementation.

## Aims and study hypotheses

The aim of the neuroimaging sub-study is to investigate neurochemical, structural and functional changes that may underpin participants’ cognitive performance due to intervention. It is hypothesised that there will be a positive correlation between brain neurochemicals and cognitive performance measures. It is also expected that there will be an improvement in cognitive performance and a change in the concentration levels of neurochemicals after 3 and 12 months intervention with *Bacopa monnieri* (CDRI08)*,* Pycnogenol*,* or a combined antioxidant formula, when compared to placebo.

The aim of the gut microflora sub-study is to investigate the effect of dietary supplements with gut microbial composition and how this composition relates to mood, cognitive performance and other health measures. It is hypothesised that the interventions will correlate with positive change in the gastrointestinal microbial composition. It is expected that a positive change in gut microbial composition will correlate with improved mood, better cognitive performance measures and other health measures included in the study.

## Design and methodology

### Design

ARCLI is a randomised, double-blind, placebo-controlled, 4-arm parallel-groups clinical trial with participants randomised to receive *Bacopa monnieri* (CDRI08)*,* Pycnogenol*,* a combined antioxidant formula, or placebo for 12 months.

### Centres

ARCLI is being conducted at the Centre for Human Psychopharmacology, Swinburne University, Melbourne, Australia. Neuroimaging will only be conducted through the Neuroimaging Facility located at Swinburne University.

### Participants

It is expected that from the overall sample of over 400 participants recruited into the ARCLI trial, prior to treatment randomisation, ~ 80 of these eligible participants (20 per arm) will elect to take part in the recently devised neuroimaging sub-study. Participants will only be included in the neuroimaging sub-study if they fulfil eligibility requirements for the main study [see 44] and do not have any metallic implants including, for example, cardiac pacemaker, cochlea implant or suffer from claustrophobia which would render them unsafe to undergo an MRI scan. Similarly, prior to randomisation, it is expected that from the overall sample of over 400 participants recruited into the ARCLI trial, ~ 300 of these participants will provide faecal stool samples at baseline for the gut microflora sub-study. All participants will provide written informed consent. The study was ethically approved by the Swinburne University Research Ethics Committee (project number 2010/106). The trial has been registered with the Australian and New Zealand Clinical Trials Registry (ACTRN12611000487910).

### Procedure

The procedure remains consistent with the main study described in the previous manuscript [44]. The procedure for the neuroimaging and gastrointestinal microflora sub-studies is described below.

#### Neuroimaging sub-study

**Fig. 1:**
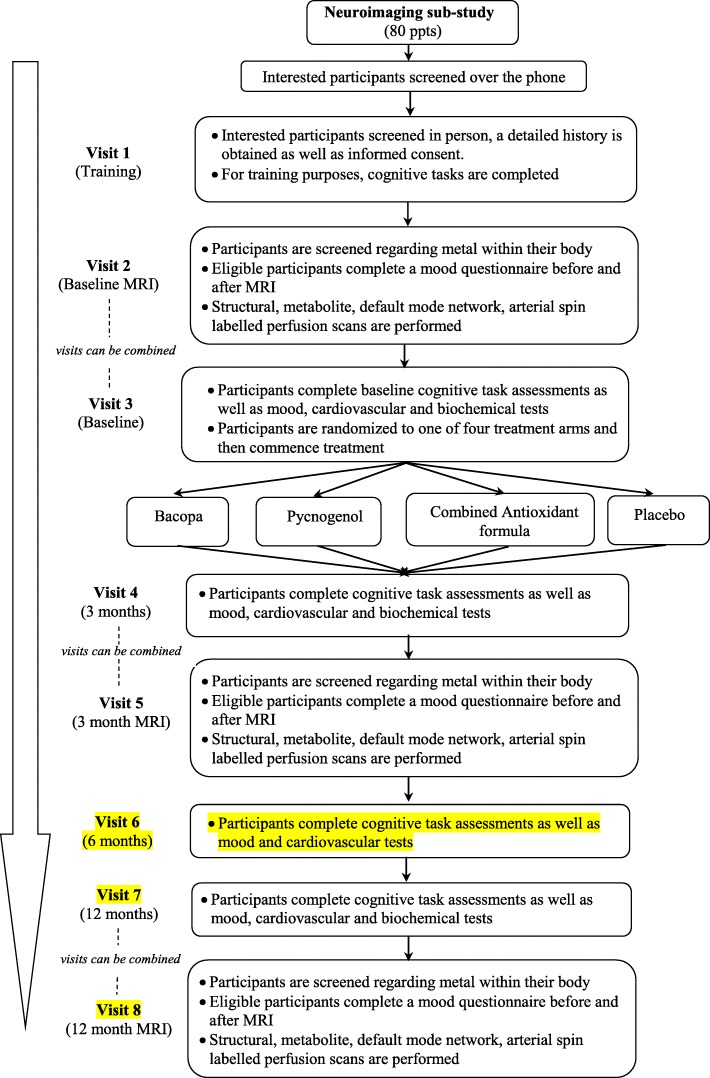
Neuroimaging Protocol Flow Diagram

Participants involved in the neuroimaging sub-study (~ 80, 20 per arm) will be required for an additional hour and a half at three time points, on either the same or separate days to their attendance at The Centre for Human Psychopharmacology, Swinburne University of Technology, for the main component of the trial [main protocol refer to 44; see Fig. 1 .integrated flow diagram]. Additional screening for eligibility to undergo MRI scans will be conducted to ensure participant safety with respect to any metallic implants within this environment. Participants will be asked to complete the “trait” and “state” assessments of the Spielberger State-Trait Anxiety Inventory prior to the MRI scans to assess a baseline anxiety level [45]. This scale is commonly used to measure fluctuating levels of anxiety. The “state” portion of the measure will be completed again, after the MRI scan, to measure participant’s anxiety levels. With the participant lying in the scanner, different imaging techniques will be utilized to look at brain structure, brain metabolite levels, blood flow and resting state connectivity.

As part of the new neuroimaging sub-study, neuroimaging measures will be collected between the training day (Visit 1) and baseline (Visit 2) assessments, and no later than a week after the 3 month (Visit 4) and 12 month assessments (Visit 7). Where possible, neuroimaging visits can be conducted on the same day as the main study assessments. Please refer to the neuroimaging protocol flow diagram, Fig. 1.

MRI scans will be collected using a Siemens 3 Tesla Tim Trio MRI scanner (Erlargen, Germany) located at the Neuroimaging Facility of Swinburne University of Technology, Hawthorn, Australia. For all participants, a high resolution (1x1x1 mm) T1-weighted structural scan will be acquired as a reference image for some of the following sequences (MRS) as well as to determine any structural changes in response to the intervention. Scanning in a resting state condition, with eyes open, looking at a fixation cross will occur to investigate intrinsic brain activity and functional networks (eg., default mode network (DMN)), using a standard echo planar imaging sequence (TE/TR = 30/2500, 3.5 × 3.5 × 3 mm, 40 slices, 144 volumes) sensitive to blood-oxygen-level-dependent contrast. Single voxel ^1^HMRS will be conducted at two regions of interest: left dorsolateral prefrontal cortex (LDLPFC; 15x12x15 mm); and posterior cingulate cortex (PC; 20x20x20 mm) using standard PRESS sequence (TE/TR = 30/2000, Ave (PC) = 124, Ave (LDLPFC) = 192, followed by two un-water supressed MRS scans at the same two ROIs to estimate the water signal. A separate MRS scan using a modified MEGA-PRESS sequence (TE/TR = 131/2000, Ave = 160, edit Pulse Frequency/Bandwidth =4.56 ppm/44 Hz) at the PC region will then be conducted to allow us to investigate oxidative stress in vivo by investigating GSH levels. An un-water-suppressed scan will then be performed with identical location and setup as above, with 12 averages, to estimate the water signal. Following MRS, to investigate the brain’s tissue perfusion, a 5-min arterial spin labelled perfusion sequence (TE/TR = 13/3000, Inversion time 1/2 = 700/1500 ms, FOV = 220 mm, 3.4 × 3.4 × 5 mm, 14 slices, with 1.25 m gap) will be performed. Participants will be in the scanner for the duration of 1 h.

#### Gastrointestinal microflora sub-study

**Fig. 2:**
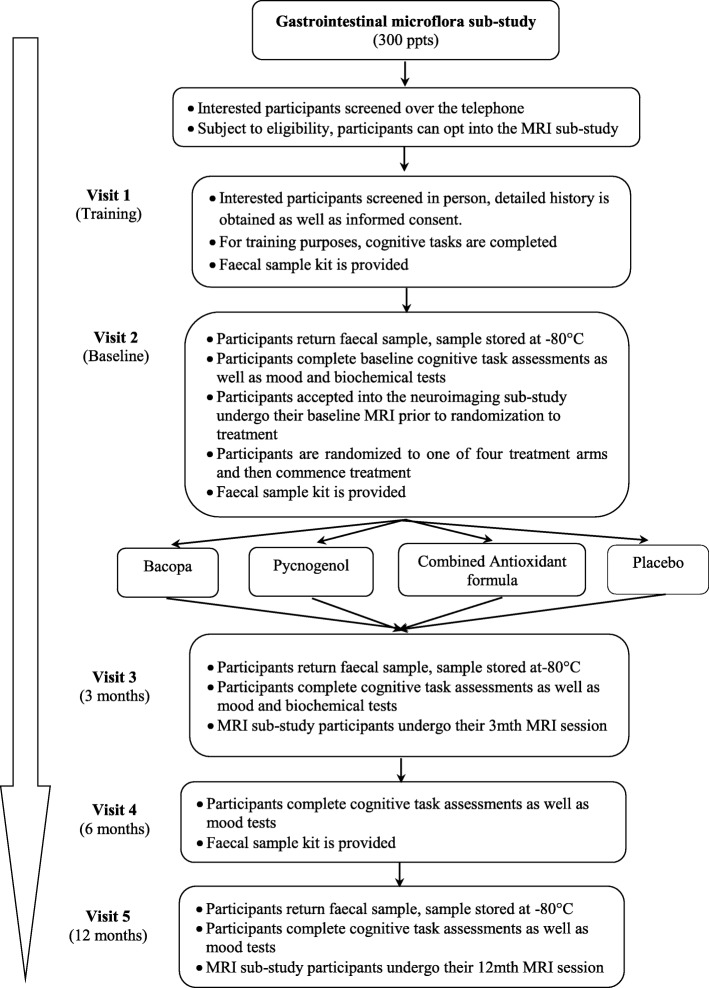
Gastrointestinal Protocol Flow Diagram

A sub-set of participants (~ 300) will be asked to supply faecal samples at baseline, 3, and 12 months for gastrointestinal microbiota analysis, please refer to the gastrointestinal microflora protocol flow diagram, Fig. 2. For these visits participants will be required to collect samples using collection vessels provided by the research team (NHS approved Easy Sampler collection kit supplied by Coverthem Limited). Samples will be required to be placed in a sealable plastic bag (provided to the participant) with a pre-frozen ice pack (frozen in advance by the participant) and stored in a freezer prior to returning to the Centre for Human Psychopharmacology, Swinburne University, at their next visit. Upon receipt of the faecal sample, the container will be stored at − 80 °C until microbiota analysis will be carried out by utilising deep next-generation shotgun sequencing [34] of DNA extracted from collected faecal samples.

## Discussion

ARCLI commenced in 2012 and will be completed in late 2019. This is a long duration for any clinical trial but is consistent with the difficulties of conducting a large trial recruiting healthy older participants. Our exclusion and inclusion criteria [see 44] are comprehensive in order to only enrol participants without other medical (physical or psychiatric) conditions that could have an impact on cognitive and brain performance. The last participant was recruited in October 2018. Taking into account the possible rescheduling/cancelling of visits we anticipate the study will finish in November 2019. More than 400 participants will have been randomized into this trial by October 2019 and given the large array of measures ranging from cognitive, psychological, diet, sleep, cardiovascular, genetic, oxidative stress, inflammatory, biochemistry, mood and well-being, and neuroimaging amongst many others, ARCLI will constitute one of the most scientifically rich studies to examine the biological basis of cognition at baseline and how two evidence-based interventions influence these variables over multiple time points (3,6 and 12 months). This information may help us develop new targets to improve cognitive and brain health in the elderly-these may be interventions that directly challenge parameters that we show are related to cognition at baseline or provide evidence of efficacy for our current interventions in terms of improving cognition or any of our outcome variables. As the last wave of intervention data is being collected, our team has started writing papers on the relationship between biological parameters and cognition (e.g. the relationship between oxidative stress and cognition [46] or oxidative stress and processing speed [47]. The baseline data is a rich and invaluable source of information. We expect more than 30 baseline papers to be written before the intervention data is finalised in August 2019. The addition of the microbiome and neuroimaging data using Magnetic Resonance Spectroscopy (MRS) is particularly exciting given the rich battery of cognitive measures used in ARCLI both at baseline and again at 3 and 12 months. Researchers interested in testing relationships between cognition and other variables collected at ARCLI would be welcome to collaborate on papers with our team.

There are some methodological considerations in conducting a trial of this size. The rigorous inclusion/exclusion criteria for participation in this study is limited to those who are healthy within the age range of 60-75 yrs. where it may be challenging to recruit the healthiest within this cohort, however, with the availability of social media avenues to advertise the trial, we have been able to broaden the reach of the study to assist in participant recruitment. Furthermore, future studies could adapt this protocol to different cohorts to investigate the outcomes of the supplements to broaden the generalisability of the population. The addition of a neuroimaging component to the trial adds an extra time commitment for participants who elect to undergo MRI scans. Prior to commencing recruitment for this sub-study we decided it would be best to offer participants the option of combining their MRI scan on the same day they complete their cognitive, mood, cardiovascular and biochemical assessments. Offering flexibility and being able to split the visits if required, may assist in less attrition rates for such a long and involved study and reduce study day fatigue. For the gastrointestinal microflora sub-study the composition of the gut microflora can be altered after probiotic and prebiotic use, increasing the growth of beneficial bacteria associated with health promotion [48]. On the other hand, antibiotics have been found to decrease intestinal microbial communities [49]. While at each study visit any changes to the participant’s medication and health status is recorded, this information is reliant upon participant recall and self-report if at any stage in the preceding 3mths they took this type of medication. It might be that despite every effort to record medication use there may be instances where study staff or researchers are unaware of this type of medication use when participants provided their stool sample. Future studies might consider providing participants with a diary to record this information and for it to be handed in at each visit.

## Conclusions

It is possible that the trajectory of ageing is malleable and that nutraceutical supplements that work on the antioxidant pathways may reduce brain and cognitive ageing. Such interventions may be a cost efficient and practical method for older adults to optimise their quality of life with advancing age. The results of this study may assist to elucidate the relationship between less known in vivo brain and gut variables in relation to cognition as well as changes in these variables due to the interventions.

### Trial status

The trial is currently recruiting.

## Abbreviations

^1^HMRS: Proton magnetic resonance spectroscopy
ANZCTR: Australia and New Zealand Clinical Trials Registry
ARCLI: Australian Research Council Longevity Intervention
Cho: Choline
Cre: Creatine
DMN: Default mode network
GLX: Glutamate & Glutamine
GSH: Glutathione
MEGA-PRESS: MEshcher-GArwood-Point RESolved spectroscopy
mI: Myo-inositol
MRI: Magnetic resonance imaging
MRS: Magnetic resonance spectroscopy
NAA: N-aceytl aspartate
PRESS: Point resolved spectroscopy
RCT: Randomised controlled trial
